# Preparation of Bamboo-Like Carbon Nanotube Loaded Piezoresistive Polyurethane-Silicone Rubber Composite

**DOI:** 10.3390/polym13132144

**Published:** 2021-06-29

**Authors:** Mohammed Nabeel, Miklós Varga, László Kuzsela, Ádám Filep, Béla Fiser, Béla Viskolcz, Mariann Kollar, László Vanyorek

**Affiliations:** 1 Institute of Chemistry, University of Miskolc, 3515 Miskolc, Hungary; mohamedxl2006@gmail.com (M.N.); miklos568@gmail.com (M.V.); kemfiser@uni-miskolc.hu (B.F.); 2 Ministry of Science and Technology—Materials Research Directorate, Baghdad 10011, Iraq; 3 Institute of Materials Science and Technology, University of Miskolc, 3515 Miskolc, Hungary; femkuzsy@uni-miskolc.hu; 4 Institute of Metallurgical and Foundry Engineering, University of Miskolc, 3515 Miskolc, Hungary; femfilep@uni-miskolc.hu; 5 Ferenc Rákóczi II. Transcarpathian Hungarian College of Higher Education, 90200 Beregszász, Transcarpathia, Ukraine; 6 Institute of Ceramics and Polymer Engineering, University of Miskolc, 3515 Miskolc, Hungary; femmaja@uni-miskolc.hu

**Keywords:** nitrogen-doped bamboo-shaped carbon nanotube, N-BCNT, PU, nanofiller, piezoresistive

## Abstract

In this study, a novel technology is reported to prepare a piezoresistive polyurethane-silicone rubber nanocomposite. Polyurethane (PU) foam was loaded with a nitrogen-doped bamboo-shaped carbon nanotube (N-BCNT) by using dip-coating, and then, impregnated with silicone rubber. PU was used as a supporting substrate for N-BCNT, while silicone rubber was applied to fill the pores of the foam to improve recoverability, compressive strength, and durability. The composite displays good electrical conductivity, short response time, and excellent repeatability. The resistance was reduced when the amount of N-BCNT (0.43 wt %) was increased due to the expanded conductive path for electron transport. The piezoresistive composite has been successfully tested in many applications, such as human monitoring and finger touch detection.

## 1. Introduction

Flexible piezoresistive pressure sensors (FPPSs) have received great attention in recent years due to their high sensitivity, controllable geometry, and easy signal collection [1,2]. Furthermore, piezoresistivity is widely utilized [3,4,5,6] due to its capacity for low-pressure detection, short response times [7], excellent durability, and lower cost [8] compare to other sensor types, such as capacitive [9,10,11,12] or transistor-based systems [13,14]. FPPSs have many important applications in the materials engineering field, such as wearable electronics [15] and electronic skin [16], which can be applied to health monitoring [17]. As a response to the mechanical forces imposed on the FPPS material its resistivity will change [18].

It is essential to select proper materials with unique structures to prepare FPPSs with specific functions. Carbonaceous nanofillers such as carbon nanotubes (CNTs) [19], graphene [20,21], and carbon black [22,23] have been the focus of attention in the field of sensing materials development. The remarkable electrical conductivity and nanostructured fibrous appearance of carbon nanotubes (CNTs) makes them more than suitable for application as additives in pressure sensors [24]. The electronic features of CNTs can be fine-tuned by incorporating heteroatoms into the graphitic structure (e.g., nitrogen-doping) [25,26,27]. The structure of the CNTs may also be modified by the incorporation of nitrogen atoms, leading to N-doped bamboo-shaped carbon nanotubes (N-BCNT). Nitrogen-doped carbon materials are widely used in several applications, such as lithium-ion batteries, fluorescent bioimaging, and supercapacitors [28,29,30]. The incorporation of N atoms can easily be realized through the application of nitrogen-containing carbon sources (e.g., different amines, or a mixture of ammonia and olefins) during CCVD (catalytic chemical vapor deposition) synthesis [31,32,33,34,35]. In the nitrogen-doped carbon nanotubes, four types of nitrogen atoms can be identified: pyridinic, pyrrolic, quaternary, and oxidized pyridinic [36]. By comparing the tunnelling spectra of N-BCNTs with multiwalled carbon nanotubes (MWCNTs), the N-doped nanotubes have an additional band at ∼0.18 eV, while in the case of MWCNTs, the valence and conduction band appear symmetric about the Fermi level [37]. This indicates that conducting behaviour of the nitrogen-doped carbon nanotubes is similar to metals [28,38,39,40]. The electrical conductivity of N-BCNTs can be fine-tuned by modifying the incorporation types and quantity of nitrogen. This can be realized by the modification of the CCVD synthesis parameters (e.g., temperature, type of carbon source, catalyst) [41,42,43,44].

Porous polymer structures, such as polydimethylsiloxane (PDMS) [45,46], polyvinylidene fluoride (PVDF) [47], and polyurethane (PU) [48,49] can be used as the matrix for the conductive nanofiller materials to prepare FPPSs. These polymeric materials act as supporting substrates for the nanofiller to achieve a mechanically flexible pressure sensor [50]. PU, with its high porosity and low elastic modulus [51], provides a promising matrix material for piezoresistive sensors. The manufacturing of polyurethane-based FPPSs involves the use of dip-coating in solutions dispersed with nanofiller materials. Using this approach, Huang et al. obtained a CNT/PU-based pressure sensor which exhibited high reproducibility and reversibility [52]. Tewari et al. incorporated CNT and rGO hybrid nanofillers to reinforce polyurethane, demonstrating that the material has a pressure sensing capability across a wide range [53]. Zhong et al. showed that their PU-based system has a pressure sensitivity of 0.17 kPa^−1^ and 0.005 kPa^−1^ under 0–6 kPa and 6–25 kPa, respectively. Li et al. were able to obtain a sensitive piezoelectric pressure sensor with quick response time (19 ms) by mixing MXene with polyurethane [50].

Two approaches have been used to create PU-based FPPSs. In the first approach the nanofiller is dispersed on the PU foam through a dip-coating process [50,52,54,55,56,57]. However, the mechanical properties of the PU skeleton (15 kPa at 60% strain) are not appropriate for wide range detection [58], because as the pressure applied to the sensors increases, the flexibility of the foam progressively decreases. Thus, the contact area of the PU skeleton becomes saturated, leading to a reduction in sensitivity and non-linearity in the mechanical-resistance behaviour of the sensors in all pressure ranges [59]. In the second approach, the nanofiller is dispersed in the polymer to create the FPPS [60,61,62,63]. However, in this case, a high nanofiller concentration has to be used to achieve the electrical threshold, because of the high viscosity of the polymeric matrix.

In this study, an innovative process was developed to create flexible piezoresistive pressure sensor materials. First, CNTs have been dispersed on a PU foam skeleton using the dip-coating process and thus, any potential waste of the nanofiller has been reduced. Then, this structure has been combined with silicone rubber to achieve the final sensor system. Thus, an FPPS with high flexibility and improved resilience has been achieved.

## 2. Experiments

### 2.1. Materials

During the nitrogen-doped bamboo-shaped carbon nanotube (N-BCNT) synthesis, *n*-butylamine (C_4_H_11_N, Sigma-Aldrich Ltd., Hamburg, Germany), nickel-nitrate hexahydrate (Ni(NO_3_)_2_∙6 H_2_O, Merck Ltd., Darmstadt, Germany), magnesium-oxide (MgO, Merck Ltd., Germany), and nitrogen (99.995% purity, Messer Ltd., Budapest, Hungary) were used. PU foams were made from toluene diisocyanate (TR4040 TDI) and polyol (FFP 303) was provided by Wanhua-BorsodChem Ltd. (Kazincbarcika, Hungary) During the impregnation of the PU foams with N-BCNT, patosolv (mixture of aliphatic alcohols, 98% ethanol and 2% isopropanol, Molar Chemicals Ltd., Halásztelek, Hungary) was used as the dispersion media. Liquide silicone rubber (Silorub ds F-20, shore hardness 20 ± 5 Å, tensile strength 1.65 N/m^2^, Bondex Ltd., Qingdao, China) and the corresponding catalyst (Silorub ds K, Bondex Ltd., China) were used to fill the PU matrix.

### 2.2. Methods

#### 2.2.1. CCVD Synthesis of Nitrogen-Doped Bamboo-Shaped Carbon Nanotubes

The synthesis of the N-BCNTs was carried by using the Catalytic Chemical Vapour Deposition (CCVD) method, applying previously optimized synthetic parameters [33]. Nickel containing (5 wt %) a magnesium oxide (2.00 g) catalyst was placed into a quartz reactor in a tube furnace, which was heated up to 750 °C. The time of synthesis was 20 min, the dosing speed of the carbon source (*n*-butylamine) was 6 mL h^−1^, while the flow rate of the carrier gas (nitrogen) was 100 mL min^−1^. The production cycle was repeated ten times and then, the N-BCNT sample was purified, and the catalytic mixture (magnesium oxide and nickel) was removed by using concentrated hydrochloride acid (36 wt %). The purity of the sample was determined by thermogravimetric analysis (TGA).

#### 2.2.2. Preparation of the Carbon Nanotube-Loaded Piezoresistive Polyurethane-Silicone Rubber Composite

**Figure 1:**
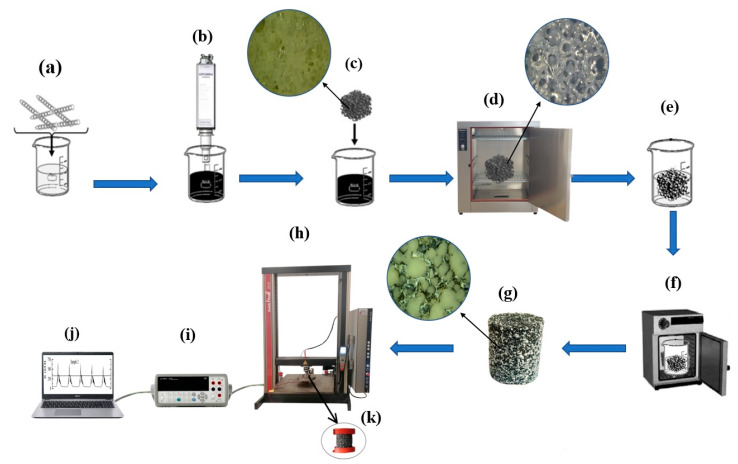
Schematic representation of the preparation and testing of the N-BCNT/PU-silicone rubber piezoresistive nanocomposite. (**a**) Mixing N-BCNT with alcohol. (**b**) Dispersion of N-BCNT in alcohol by ultrasonication. (**c**) Dipping the PU samples into the N-BCNT solution and (**d**) drying the N-BCNT/PU system. (**e**,**f**) Vacuum impregnation of the N-BCNT/PU samples with silicone rubber. (**g**) Cutting the N-BCNT/PU-silicone rubber nanocomposite samples. The pressure response of the samples (**k**) has been tested by using a (**h**) tensile test device, (**i**) data recorder, and (**j**) computer to collect the data.

The piezoresistive composite preparation was carried out in multiple steps (Figure 1). First, the PU foam was prepared by mixing 11.9 g of TR4040 isocyanate and 20.0 g of FFP 303 polyol by using a shear mixer for 10 s at 5000 rpm and room temperature. After that, the PU foam was cut into rectangular pieces (45 mm × 42 mm × 38 mm). Then, three different amounts of N-BCNT (0.1, 0.2, and 0.3 g) were dispersed in patosolv (150 mL) by Hielscher UIPHdt1000 tip homogenizer (340 W/19.42 kHz) for 8 min. Thereafter, the PU samples were dipped into the N-BCNT solutions, and then, the treated foam samples were dried at 105 °C to evaporate the solvent and to obtain the N-BCNT/PU systems. The dipping and drying process was repeated three times to maximize the amount of N-BCNT absorbed by the foam samples. In the next step, the N-BCNT/PU systems were vacuum impregnated with silicone rubber to fill the pores of the foam as much as possible. Thus, N-BCNT/PU-silicone rubber nanocomposite samples were obtained (Table 1), which were cut into cylindrical shapes before examination.

## 3. Characterization Techniques

The synthesized carbon nanotubes were examined by high-resolution transmission electron microscopy (HRTEM) using an FEI Technai G2-20X Twin machine (acceleration voltage: 200 kV). The sample preparation was carried out by dropping the aqueous suspension of the samples onto a copper grid (300 Mesh, only carbon from Ted Pella). The silicone rubber-loaded N-BCNT/PU foams were studied by high-resolution scanning electron microscope (SEM) as well by applying a Helios G4 PFIB CXe (Thermo Fisher Scientific, Waltham, MA, U.S.) instrument and using carbon tape for sample preparation. During sample preparation, the foams were coated with a gold sputtering. A Zeiss Discovery V12 stereo microscope was also used to examine the foam samples.

After acidic purification, the carbon content (purity) of the N-BCNTs was determined by thermogravimetric analysis (TGA) using a Netzsch Tarsus TG 209 thermo-microbalance. TGA measurements have been carried out in a mixed atmosphere (nitrogen (4.5) and oxygen (5.0)), where the flowrate was set to 6 mL∙min^−1^ and 14 mL∙min^−1^ for O_2_ and N_2_, respectively. The purity of the carbon nanotubes, i.e., carbon content, was determined by thermogravimetric analysis (TGA). Combustion in pure oxygen was deemed too fast; thus, a mixture of nitrogen and oxygen was used to burn the carbon content of the samples. The heating rate was 10 °C min^−1^, in the 35–800 °C temperature range.

The surface functional groups of the N-BCNTs were identified by Fourier-transform infrared spectroscopy (FTIR) with a Bruker Vertex 70 spectrometer. The prepared N-BCNT sample (2 mg) was added to 250 mg spectroscopic potassium bromide, and after homogenization a pellet was created which was used in the measurements carried out in transmission mode.

Malvern Nano Zs equipment was used to measure the zeta (electro-kinetic) potential of the N-BCNT sample, and the electrophoretic mobility was determined by using the Smoluchowski equation. For this purpose, the N-BCNT sample (2 mg) was dispersed by using an ultrasonic bath in 250 mL distilled water.

The chemical binding types of the incorporated nitrogen atoms have been determined by X-ray photoelectron spectroscopy (XPS) using a SPECS Phoibus 150 MCD nine analyser.

Micro CT measurements with YXLON FF35 equipment (microfocus X-ray tube, transmission beam, acceleration voltage: 90 kV, Al filter: 0.5 mm, voxel size: 15.6 µm) have been carried out to determine the degree of incorporation of the silicone rubber into the pores of the PU foam. The pores were analysed by using the porosity analysis/foam structure analysis modules of the VG Studio software after applying adaptive gaussian filtering.

## 4. Results and Discussion

### 4.1. Characterization of the N-Doped Carbon Nanotubes

**Figure 2:**
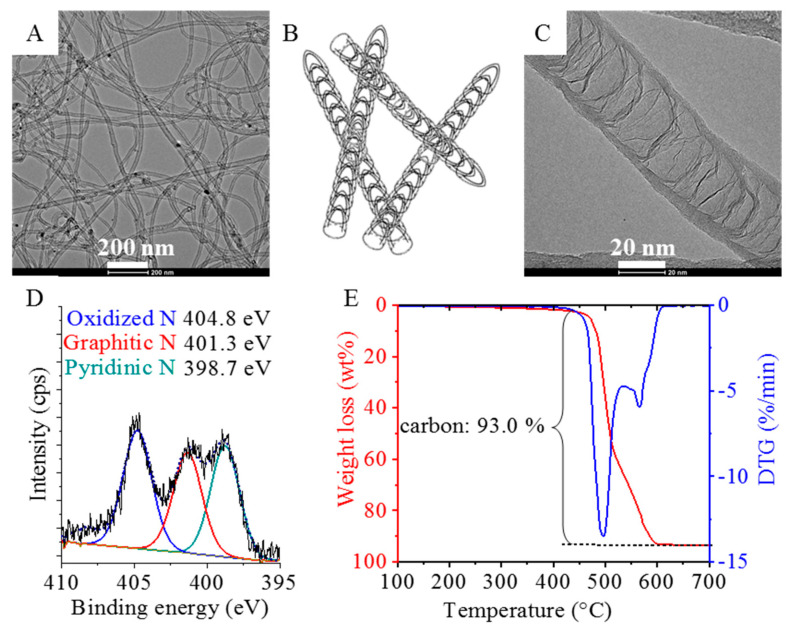
(**A**) TEM image, (**B**) schematic illustration, (**C**) HRTEM image, (**D**) XPS spectrum, and (**E**) TGA–DTG curves of the N-BCNT sample.

The fibrous structure of the carbon nanotubes can be seen on the HRTEM image of the purified N-BCNT sample (Figure 2A). The average diameter of the outer tube is 19.7 ± 6.5 nm. Catalyst-related contamination is not visible next to the nanotubes. The hemi-spherical fullerene-like building blocks of the N-BCNTs are also visible on the images (Figure 2C).

The FTIR analysis shows that peaks originating from the CNT structure can be found at 1634 cm^−1^, 1665 cm^−1^, 2898 cm^−1^, and 2902 cm^−1^ wavenumbers, and these can be associated with the νC=C, νC=O bands, and the symmetric and asymmetric C–H stretching vibrations, respectively. Furthermore, oxygen-containing functional groups (e.g., hydroxyl (–OH)) are present on the walls of the N-BCNTs, which makes it easier to disperse them in liquid phase (Figure S1A). The stretching vibration mode of the C–O bonds have been identified at 1254 cm^−1^. The bending and stretching vibration modes of the OH groups are found at 1393 cm^−1^ and 3448 cm^−1^, respectively. The hydroxyl groups on the surface of the N-BCNTs can be deprotonated in aqueous media, which leads to negative surface charge, which was proved by zeta (ζ) potential measurements (Figure S1B). The ζ potential of the aqueous dispersion of the N-BCNT sample was negative (ζ = −21.8 mV) and thus, it formed a stable dispersion. The electronegative heteroatom (nitrogen) incorporation and the presence of oxidized nitrogen species (based on XPS measurement see below) also increased the negative character of the N-BCNTs and enhanced the wettability.

The nitrogen incorporation was confirmed by XPS measurements. By the deconvolution of the N 1s band, the C-N binding types in the N-BCNT structure have been identified (Figure 2D). Three bands can be located on the XPS spectrum at 404.8 eV, 401.3 eV, and 398.7 eV, which correspond to the oxidized (pyridine N-oxide), graphitic, and pyridinic nitrogen, respectively. By changing the nitrogen content, the conductivity of the nanotubes can be modified [41,42].

The TGA analysis shows that the total carbon content (purity) of the N-BCNT was 93 wt % (Figure 2E).

### 4.2. Morphological Characterization of the N-BCNT/PU System

The structure and morphology of the N-BCNT/PU system have also been examined by optical microscopy and scanning electron microscopy (Figure S2A–D). The high quantity of micro-pores in PU has been validated with optical microscopy as well, and it can be seen that the surface area is high and fully covered by N-BCNTs (Figure S2A). The impregnation of the silicone rubber inside the PU foam structure can also be seen on the optical microscopy images (Figure S2B). PU acts as supporting substrate for the N-BCNT, which is responsible for creating a conductive path inside the insulator silicone rubber. The SEM images confirmed that the PU structure has been coated successfully with N-BCNTs (Figure S2D). The PU skeleton displayed a wrinkled structure due to the presence of N-BCNT, which indicated that the nanotubes were attached to the foam through the dip-coating process. The N-BCNTs serve as a 3D conductive network in the PU which will change its electrical features when pressure is applied to the system.

**Figure 3:**
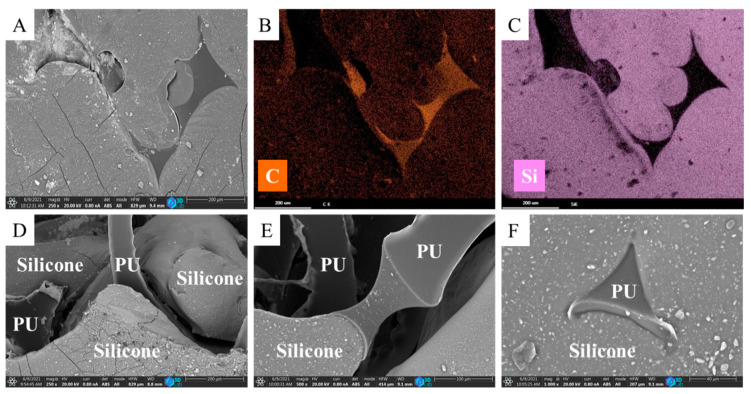
SEM images of the N-BCNT/PU-silicone rubber sample (**A**), element maps of the carbon from the PU matrix (**B**) and the silicone of the silicone rubber (**C**). Highlighted areas of the silicone rubber and PU in the N-BCNT/PU-silicone rubber sample (**D**–**F**).

The N-BCNT/PU-silicone rubber was examined by SEM as well and cross-section SEM images have been created (Figure 3A–F). The silicone rubber filled the pores of the N-BCNT/PU foam (Figure 3A). The silicone rubber and the N-BCNT/PU foam can be clearly distinguished in two layers on the element mapping of the nanocomposite (Figure 3B,C). The red colour represents carbon (from N-BCNTs and PU), while the purple represents silicone. The surface of the PU is partially covered by silicone rubber (Figure 3A–F), whose primary role is to improve the structural stiffness of the composite. All in all, good adhesion was detected among the constituents of the nanocomposite, which is clearly observed in the performance of the samples under cyclic load.

### 4.3. X-ray Micro Computed Tomography (CT) and Optical Microscopy Characterization of the PU and N-BCNT/PU-Silicone Rubber

**Figure 4:**
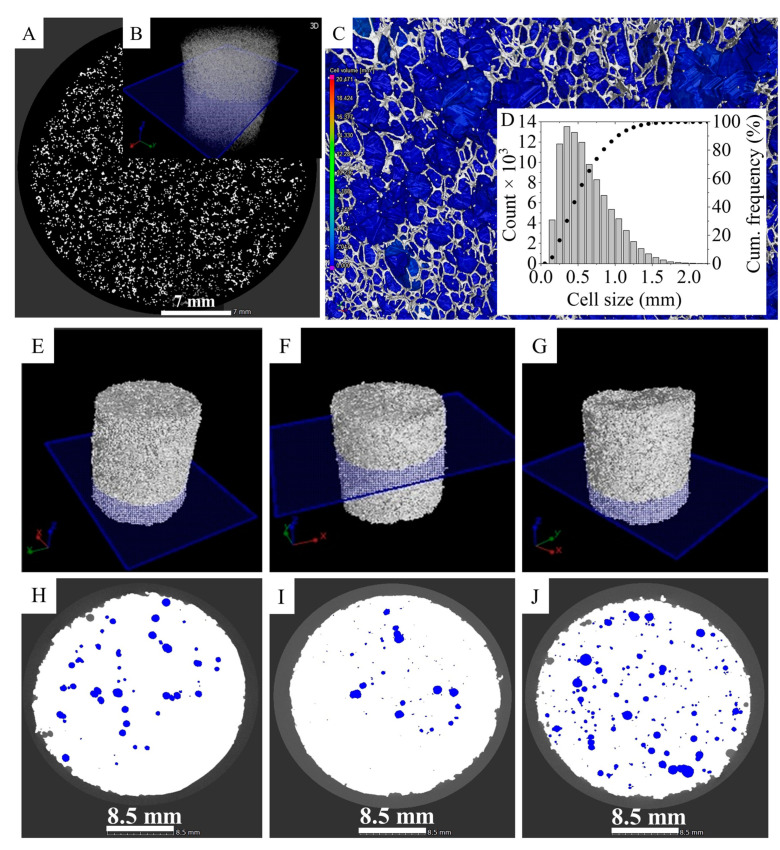
(**A**) Micro-CT image of the PU foam. (**B**) The mapping plane’s location in the sample. (**C**) Cell structure (blue: pores, grey: walls) and (**D**) size distribution. Micro-CT images of the N-BCNT/PU-silicone rubber nanocomposites. Position of the mapping plane and CT scan of sample 1—(**E**,**H**), sample 2—(**F**,**I**), and sample 3—(**G**,**J**). The white and blue areas are filled and not filled with silicone rubber, respectively.

A micro-CT scan was used to map the cell structure of the pure PU foam. The applied scan plane was 8 µm on the upper half portion of the sample. The norm of the mapping plane is parallel to the sample’s long axis. The scan indicates that the majority of the pores in the PU were open and available to filled with silicone rubber (Figure 4A). Most of the pores (90%) are >50 µm, but <1000 µm, while the average diameter of the cells is 620 ± 329 µm. This indicates that the silicone rubber can easily penetrate into the PU foam. The N-BCNT/PU-silicone rubber nanocomposites were also characterized by using micro-CT scans (Figure 4E–J). The PU foam was least filled in case of sample 3 (air volume 5.6 vol%), while sample 2 was the one which was filled the most with silicone rubber (air volume 1.6 vol%). In the absence of silicone rubber, the nanotubes could be removed unintentionally from the N-BCNT/PU system when pressure was applied, as only van der Waals forces keep them together. However, when the N-BCNT-coated PU is impregnated with silicone rubber, the position of the nanotubes will be fixed and the structural integrity will be preserved during mechanical impact.

### 4.4. Electrical and Piezoresistive Properties

**Figure 5:**
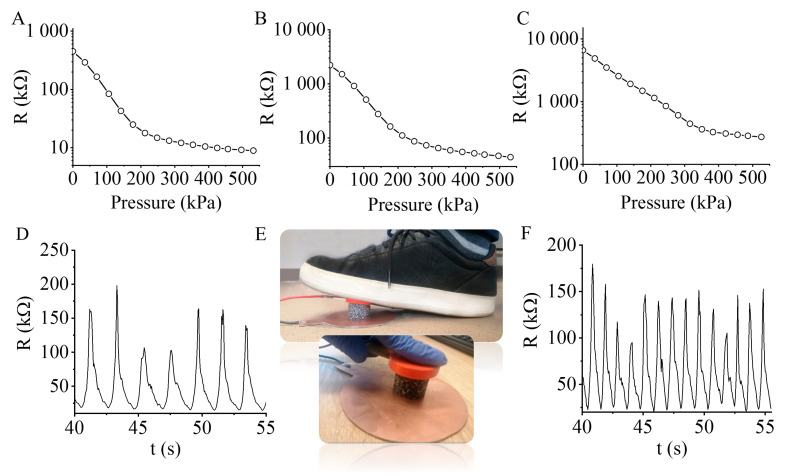
Resistance (kΩ) versus pressure (kPa) of the prepared N-BCNT/PU-silicone rubber systems: (**A**) sample 1, (**B**) sample 2, and (**C**) sample 3. Motion detection results (**D**) and its test (**E**), and signals of the finger touch detection (**F**) with the developed N-BCNT/PU-silicone rubber nanocomposite.

The electrical resistance of the N-BCNT/PU-silicone rubber nanocomposites is studied in response to the applied pressure (Figure 5). The load was measured by a universal electromechanical testing machine and the resistance was measured by multimeter which was synchronised with the data. All measurements were carried at room temperature. The prepared N-BCNT/PU-silicone rubber nanocomposite is a quasi-isotropic material in macroscale, as proved by the X-ray micro computed tomography measurements. The composite underwent a uniaxial compression test (Figure 5E) and the results suggest that all the 1D properties can be expanded to 2D and 3D. Thus, the uniaxial measured properties can be considered as 3D properties.

It was found that the resistance decreased when the applied pressure increased. A higher N-BCNT concentration led to the lower initial resistance of the nanocomposite. In the case of sample 1 and sample 3 the N-BCNT concentration is 0.43 wt % (Figure 5A) and 0.13 wt % (Figure 5C), while the initial resistance for these systems is 450 kΩ and 6500 kΩ, respectively. In the elastic zone, where the pressure is 0–80 kPa, the resistance decreases linearly with pressure in each case. This linear range (80 kPa) is wide compared to other piezoresistive PU composites, whereas this was 2.5, 10, and 40 kPa by using graphene oxide and MWCNTs [18,53,64]. Furthermore, it has to be noted that this wide linear range was obtained by using only <0.5 wt % N-BCNTs content. The plateau zone is between 80 kPa and 210 kPa, where the pressure increases gradually with a flattening in the curve. In the compaction zone (>210 kPa), the pressure rises dramatically, because the conductive N-BCNTs get too close to each other within the PU skeleton.

The N-BCNT/PU-silicone rubber system (sample 1) is well usable in motion and finger touch detection (Figure 5D–F). The N-BCNT/PU-silicone rubber nanocomposites are applicable also to movement detection due to their excellent piezoresistive and compressibility features (Figure 5D). To measure motion detection (or step-counter), the piezoresistive composite was placed between two electrodes, and the resistance signals were collected during the loading and unloading pressure sequence (Figure 5E). The peak amplitudes were dependent on the amount of pressure applied on the nanocomposite by the foot or finger recorded for each movement. Thus, the nanocomposites’ ability to sense different kind of movements has been proven.

The applicability and excellent recoverability of the nanocomposite in such a wide pressure range is due to the impregnation of silicone rubber inside the N-BCNT/PU system (Figure S3). The developed nanocomposite is more suitable for applications at higher pressures compare to systems with unfilled pores, which are limited to a maximum pressure of 60 kPa due to the low compression strength of the foam-based polymeric materials [51,53,54].

**Figure 6:**
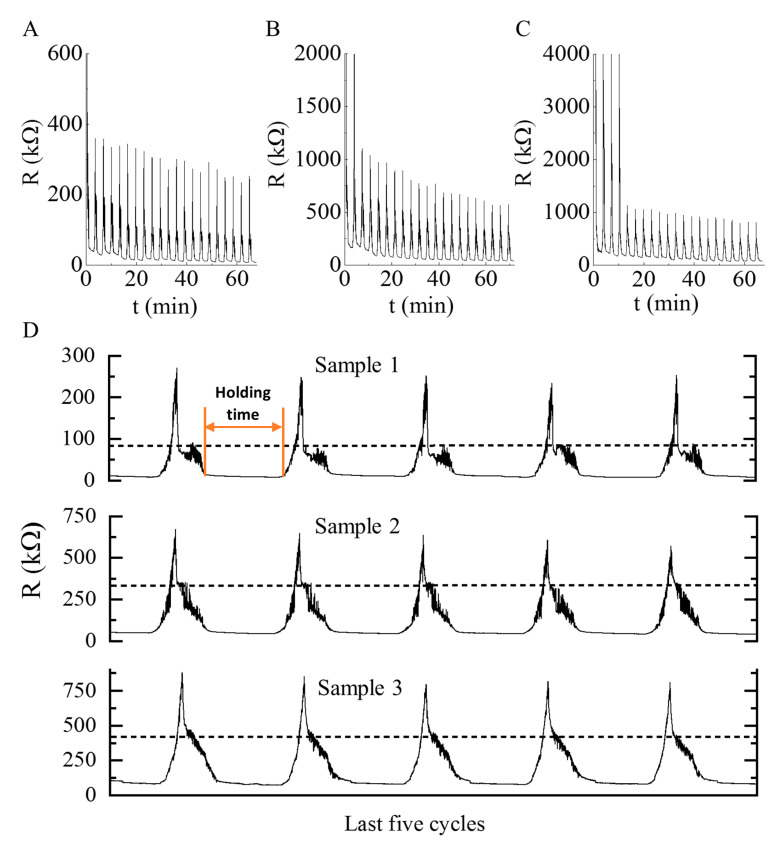
Cyclic pressure tests of (**A**) sample 1, (**B**) sample 2, (**C**) sample 3 flexible piezoresistive N-BCNT/PU-silicone rubber nanocomposites, and (**D**) recoverability tests of the samples.

Cyclic experiments were conducted to study the repeatability and recoverability of the nanocomposites (Figure 6). The pressures were ramped up from 10% to 30% with a holding time of two minutes after each cycle. The samples were tested in twenty compressive loading and unloading. The cycles and peak amplitudes were uniform and stable after a few cycles, indicating that the developed piezoresistive nanocomposite has an excellent ability to detect signal outputs with different pressures. The peaks were stable from the start of the experiment in case of sample 1, which contains 0.43 wt % N-BCNT. However, in case of sample 2 and sample 3 with 0.21 wt % and 0.13 wt % N-BCNTs, stable peaks were achieved only after two and four cycles, respectively. The fluctuations in the first cycles when N-BCNT was present in the system at a lower concentration may be related to nano- or microcrack formation in the nanotube layers of the composite [65]. The developed piezoresistive nanocomposite is reliable, and its recoverability is also proved (Figure 6D). As can be seen, the magnitude of the maximum resistance remained unchanged even after two minutes of holding time for many cycles (Figure 6). The response time was very short for sample 1, sample 2, and sample 3, respectively. The fast response enabled the nanocomposite to detect wide variety of signals such as motions, finger touch, and knock detection (Figure 5).

Various carbon forms such as graphene, graphene oxide, multiwalled carbon nanotubes, and carbon black have already been used to make piezoresistive PU-silicone rubber composites for sensor development [18,19,20,21,22,23,65]. The carbon content in the present composites is very low (0.13–0.43 wt %). Owing to the fibrous structure of the N-BCNTs, the total volume of the PU-silicone rubber matrix is woven through by carbon nanotubes, thus less is needed than for carbon black, graphite, or graphene. Furthermore, due to the excellent properties of the composite, even this small nanotube content is still enough to reach a well detectable signal and short response time.

### 4.5. Structural Stiffness of the Pieziresistive Nanocomposite

**Figure 7:**
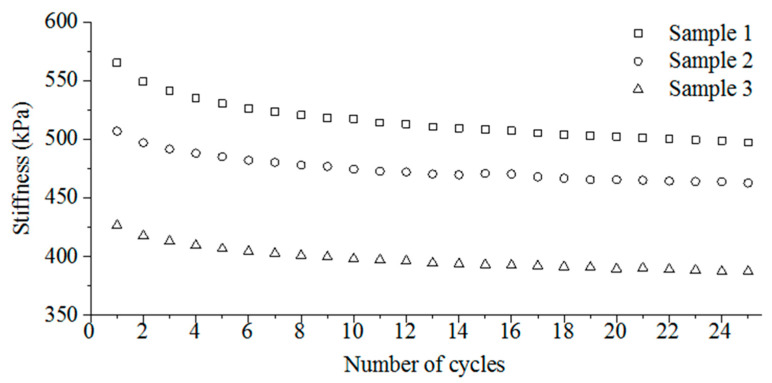
The calculated stiffness as a function of the loading cycles in the case of the N-BCNT/PU-silicone rubber nanocomposites.

The applied force was also measured besides the resistance of the samples during the compression tests (Figure 7). Based on the force needed to compress the samples up to 30%, their structural stiffness can be calculated knowing the original height and diameter of the specimens (Figure 7).

In each case, the stiffness of the samples slightly decreased as the cycles progressed (Figure 7). This proves the elastico-plastic behaviour of the developed N-BCNT/PU-silicone rubber nanocomposites.

Furthermore, the structural stiffness strongly depends on the N-BCNT content. As the N-BCNT content decreased from sample 1 to sample 3 (from 0.43 to 0.13 wt %, respectively) the structural stiffness also decreased (Table 2).

## 5. Conclusions

In this study, a novel technique to prepare flexible piezoresistive nanocomposites has been developed by dispersing N-BCNTs in PU foam followed by impregnation with silicone rubber. The developed flexible piezoresistive composite has many features, including fast response time, robustness, excellent repeatability, and a wide pressure range of application (up to 520 kPa). Nitrogen-doped carbon nanotubes have been used as conductive filler, because of their remarkable electronic behaviour. Their fibrous structure spans the entire volume of the polyurethane matrix, allowing them to act as nanometric electric cables that provide conductivity in the macroscopic dimension of the N-BCNT/PU-silicone system. The nanotubes are well dispersible in the PU matrix, and thus, a small amount is enough to reach measurable electric conductivity. However, despite all the good features of the N-BCNT/PU composite, impregnation with silicone rubber was necessary to achieve the desired properties. The prepared N-BCNT/PU-silicone rubber nanocomposite structure showed improved flexibility, mechanical resilience, and optimized durability due to the presence of silicone rubber which prevented the nanotubes from breaking apart from the polyurethane foam during pressure application tests. The developed piezoresistive nanocomposite has shown good repeatability under cyclic load, because of the excellent adhesion between its constituents. Furthermore, the resistance range can be modified by controlling the amount of nanotubes in the composite structure. Another advantage of the use of N-BCNTs as electrically conductive filler is that this type of carbon form is only necessary in small amounts to reach a well detectable signal, and short response time, compared to non-fibrous materials.

The final N-BCNT/PU-silicone rubber nanocomposite samples have also been tested in various applications and their ability to detect different pressure signals, such as motion and finger touch detection, has been proven. Therefore, this study provides an economic and reliable method to quickly and effectively produce flexible piezoresistive PU-silicone rubber composites that can be useful for a wide range of applications and immediate implementation in health care, automotive, and space industries.

## Figures and Tables

**Table 1:** Ratios of silicone rubber, PU, and N-BCNT in the prepared N-BCNT/PU-silicone rubber samples.

Samples	N-BCNT wt %	PU wt %	Silicon Rubber wt %
Sample 1	0.43	4.52	95.05
Sample 2	0.21	3.07	96.71
Sample 3	0.13	3.5	96.37

**Table 2:** Stiffness of the N-BCNT/PU-silicone rubber flexible piezoresistive nanocomposites.

	Sample 1	Sample 2	Sample 3
Average, kPa	516.0	474.9	398.2
Standard deviation, kPa	17.25	11.46	10.10
Relative standard deviation, %	3.34	2.41	2.54

## Data Availability

The data presented in this study are available on request from the corresponding author.

## Supplementary Materials

The following are available online at https://www.mdpi.com/article/10.3390/polym13132144/s1, Figure S1: FTIR spectrum (A) and zeta potential distribution in aqueous dispersion (B) of the N-BCNTs. Figure S2: Optical microscopy images. Figure S3: Schematic explanation of the effect of silicone rubber. Figure S4: Robotic arm for the injection of PU components.

## References

[1] Yang J., Ye Y., Li X., Lü X., Chen R. (2018). Flexible, conductive, and highly pressure-sensitive graphene-polyimide foam for pressure sensor application. Compos. Sci. Technol..

[2] Mei H., Zhang C., Wang R., Feng J., Zhang T. (2015). Impedance characteristics of surface pressure-sensitive carbon black/silicone rubber composites. Sens. Actuators A Phys..

[3] Narongthong J., Das A., Le H.H., Wießner S., Sirisinha C. (2018). An efficient highly flexible strain sensor: Enhanced electrical conductivity, piezoresistivity and flexibility of a strongly piezoresistive composite based on conductive carbon black and an ionic liquid. Compos. Part A Appl. Sci. Manuf..

[4] Li C., Xie J., Cordovilla F., Zhou J., Jagdheesh R., Ocaña J.L. (2018). Design, fabrication and characterization of an annularly grooved membrane combined with rood beam piezoresistive pressure sensor for low pressure measurements. Sens. Actuators A Phys..

[5] Choi W., Lee J., Yoo Y.K., Kang S., Kim J., Lee J.H. (2014). Enhanced sensitivity of piezoelectric pressure sensor with microstructured polydimethylsiloxane layer. Appl. Phys. Lett..

[6] Sharma T., Je S.S., Gill B., Zhang J.X.J. (2012). Patterning piezoelectric thin film PVDF-TrFE based pressure sensor for catheter application. Sens. Actuators A Phys..

[7] Chun S., Son W., Choi C. (2018). Flexible pressure sensors using highly-oriented and free-standing carbon nanotube sheets. Carbon.

[8] Wang G., Liu T., Sun X.-C., Li P., Xu Y.-S., Hua J.-G., Yu Y., Li S.-X., Dai Y.-Z., Song X.-Y. (2018). Flexible pressure sensor based on PVDF nanofiber. Sens. Actuators A Phys..

[9] Park S.W., Das P.S., Park J.Y. (2018). Development of wearable and flexible insole type capacitive pressure sensor for continuous gait signal analysis. Org. Electron..

[10] Yoon J.I., Choi K.S., Chang S.P. (2017). Novel means of fabricating microporous structures for the dielectric layers of capacitive pressure sensor. Microelectron. Eng..

[11] Kang M.-C., Rim C.-S., Pak Y.-T., Kim W.-M. (2017). A simple analysis to improve linearity of touch mode capacitive pressure sensor by modifying shape of fixed electrode. Sens. Actuators A Phys..

[12] Wan S., Bi H., Zhou Y., Xie X., Su S., Yin K., Sun L. (2017). Graphene oxide as high-performance dielectric materials for capacitive pressure sensors. Carbon.

[13] So H.M., Sim J.W., Kwon J., Yun J., Baik S., Chang W.S. (2013). Carbon nanotube based pressure sensor for flexible electronics. Mater. Res. Bull..

[14] Schwartz G., Tee B.C.-K., Mei J., Appleton A.L., Kim D.H., Wang H., Bao Z. (2013). Flexible polymer transistors with high pressure sensitivity for application in electronic skin and health monitoring. Nat. Commun..

[15] Zhong Y., Tan X., Shi T., Huang Y., Cheng S., Chen C., Liao G., Tang Z. (2018). Tunable wrinkled graphene foams for highly reliable piezoresistive sensor. Sens. Actuators A Phys..

[16] Chen H., Miao L., Su Z., Song Y., Han M., Chen X., Cheng X., Chen D., Zhang H. (2017). Fingertip-inspired electronic skin based on triboelectric sliding sensing and porous piezoresistive pressure detection. Nano Energy.

[17] Yue Y., Liu N., Liu W., Li M., Ma Y., Luo C., Wang S., Rao J., Hu X., Su J. (2018). 3D hybrid porous Mxene-sponge network and its application in piezoresistive sensor. Nano Energy.

[18] Yao H.-B., Ge J., Wang C.-F., Wang X., Hu W., Zheng Z.-J., Ni Y., Yu S.-H. (2013). A Flexible and Highly Pressure-Sensitive Graphene-Polyurethane Sponge Based on Fractured Microstructure Design. Adv. Mater..

[19] Zhang P., Lei S., Fu W., Niu J., Liu G., Qian J., Sun J. (2018). The effects of agglomerate on the piezoresistivity of conductive carbon nanotube/polyvinylidene fluoride composites. Sens. Actuators A Phys..

[20] Wang T., Li J., Zhang Y., Liu F., Zhang B., Wang Y., Jiang R., Zhang G., Sun R., Wong C. (2019). Highly Ordered 3D Porous Graphene Sponge for Wearable Piezoresistive Pressure Sensor Applications. Chem. A Eur. J..

[21] Kou H., Zhang L., Tan Q., Liu G., Lv W., Lu F., Dong H., Xiong J. (2018). Wireless flexible pressure sensor based on micro-patterned Graphene/PDMS composite. Sens. Actuators A Phys..

[22] Zhai W., Xia Q., Zhou K., Yue X., Ren M., Zheng G., Dai K., Liu C., Shen C. (2019). Multifunctional flexible carbon black/polydimethylsiloxane piezoresistive sensor with ultrahigh linear range, excellent durability and oil/water separation capability. Chem. Eng. J..

[23] Zhai Y., Yu Y., Zhou K., Yun Z., Huang W., Liu H., Xia Q., Dai K., Zheng G., Liu C. (2020). Flexible and wearable carbon black/thermoplastic polyurethane foam with a pinnate-veined aligned porous structure for multifunctional piezoresistive sensors. Chem. Eng. J..

[24] Wang C., Murugadoss V., Kong J., He Z., Mai X., Shao Q., Chen Y., Guo L., Liu C., Angaiah S. (2018). Overview of carbon nanostructures and nanocomposites for electromagnetic wave shielding. Carbon.

[25] Terrones M., Jorio A., Endo M., Rao A., Kim Y.A., Hayashi T., Charlier J.-C., Dresselhaus G., Dresselhaus M., Terrones M. (2004). New direction in nanotube science. Mater. Today.

[26] Jorio A., Dresselhaus G., Dresselhaus M.S. (2008). Carbon Nanotubes: Advanced Topics in the Synthesis, Structure, Properties and Applications.

[27] Belz T., Baue A., Find J., Gunter M., Herein D., Möckel H., Pfänder N., Sauer H., Schulz G., Schütze J. (1998). Structural and chemical characterization of N-doped nanocarbons. Carbon.

[28] Idrees M., Batool S., Kong J., Zhuang Q., Liu H., Shao Q., Lu N., Feng Y., Wujcik E.K., Gao Q. (2019). Polyborosilazane derived ceramics—Nitrogen sulfur dual doped graphene nanocomposite anode for enhanced lithium ion batteries. Electrochim. Acta.

[29] Qi H., Teng M., Liu M., Liu S., Li J., Yu H., Teng C., Huang Z., Liu H., Shao Q. (2019). Biomass-derived nitrogen-doped carbon quantum dots: Highly selective fluorescent probe for detecting Fe3+ ions and tetracyclines. J. Colloid Interface Sci..

[30] Du W., Wang X., Zhan J., Sun X., Kang L., Jiang F., Zhang X., Shao Q., Dong M., Liu H. (2019). Biological cell template synthesis of nitrogen-doped porous hollow carbon spheres/MnO2 composites for high-performance asymmetric supercapacitors. Electrochim. Acta.

[31] Suenaga K., Johansson M., Hellgren N., Broitman E., Wallenberg R., Colliex C., Sundgren J.-E., Hultman L. (1999). Carbon nitride nanotubulite—Densely-packed and well-aligned tubular nanostructures. Chem. Phys. Lett..

[32] Terrones M., Grobert N., Hsu W.K., Zhu Y., Hare J.P., Kroto H.W., Walton D.R.M., Kohler-Redlich P., Rühle M., Zhang J.P. (1999). Efficient route to large arrays of CNx nanofibers by pyrolysis of ferrocene/melamine mixtures. Appl. Phys. Lett..

[33] Terrones M., Grobert N., Terrones H. (2002). Synthetic routes to nanoscale BxCyNz architectures. Carbon.

[34] Lee Y.T., Kim N.S., Park J., Han J., Choi Y.S., Ryu H., Lee H.J. (2003). Temperature-dependent growth of carbon nanotubes by pyrolysis of ferrocene and acetylene in the range between 700 and 1000 °C. Chem. Phys. Lett..

[35] Glerup M., Castignolles M., Holzinger M., Hug G., Loiseau A., Bernier P. (2003). Synthesis of highly nitrogen-doped multi-walled carbon nanotubes. Chem. Commun..

[36] Matter P.H., Zhang L., Ozkan U.S. (2006). The role of nanostructure in nitrogen-containing carbon catalysts for the oxygen reduction reaction. J. Catal..

[37] Tekleab D., Czerw R., Carroll D.L., Ajayan P.M. (2000). Electronic structure of kinked multiwalled carbon nanotubes. Appl. Phys. Lett..

[38] Czerw R., Terrones M., Charlier J.-C., Blase X., Foley B., Kamalakaran R., Grobert N., Tekleab D., Blau W., Rühle A.M. (2001). Identification of Electron Donor States in N-Doped Carbon Nanotubes. Nano Lett..

[39] Golberg D., Dorozhkin P.S., Bando Y., Dong Z.-C., Tang C.C., Uemura Y., Grobert N., Reyes-Reyes M., Terrones H., Terrones M. (2003). Structure, transport and field-emission properties of compound nanotubes: CNx vs. BNCx (x<0.1). Appl. Phys. A Mater. Sci. Process..

[40] Villalpando-Paez F., Zamudio A., Elias A.L., Son H., Barros E.B., Chou S.G., Kim Y.A., Muramatsu H., Hayashi T., Kong J. (2006). Synthesis and characterization of long strands of nitrogen-doped single-walled carbon nanotubes. Chem. Phys. Lett..

[41] Lee D.H., Lee W.J., Kim S.O. (2009). Highly Efficient Vertical Growth of Wall-Number-Selected, N-Doped Carbon Nanotube Arrays. Nano Lett..

[42] Wiggins-Camacho J.D., Stevenson K.J. (2009). Effect of Nitrogen Concentration on Capacitance, Density of States, Electronic Conductivity, and Morphology of N-Doped Carbon Nanotube Electrodes. J. Phys. Chem. C.

[43] Vanyorek L., Muranszky G., Sikora E., Pénzeli X., Prekob Á., Kiss A., Fiser B., Viskolcz B. (2019). Synthesis Optimization and Characterization of Nitrogen-Doped Bamboo-Shaped Carbon Nanotubes. J. Nanosci. Nanotechnol..

[44] Fujisawa K., Tojo T., Muramatsu H., Elías A.L., Vega-Díaz S.M., Tristán-López F., Kim J.H., Hayashi T., Kim Y.A., Endo M. (2011). Enhanced electrical conductivities of N-doped carbon nanotubes by controlled heat treatment. Nanoscale.

[45] Rinaldi A., Tamburrano A., Fortunato M., Sarto M.S. (2016). A Flexible and Highly Sensitive Pressure Sensor Based on a PDMS Foam Coated with Graphene Nanoplatelets. Sensors.

[46] Michel T.R., Capasso M.J., Cavusoglu M.E., Decker J., Zeppilli D., Zhu C., Bakrania S., Kadlowec J.A., Xue W. (2020). Evaluation of porous polydimethylsiloxane/carbon nanotubes (PDMS/CNTs) nanocomposites as piezoresistive sensor materials. Microsyst. Technol..

[47] Xu D., Zhang H., Pu L., Li L. (2020). Fabrication of Poly(vinylidene fluoride)/Multiwalled carbon nanotube nanocomposite foam via supercritical fluid carbon dioxide: Synergistic enhancement of piezoelectric and mechanical properties. Compos. Sci. Technol..

[48] Ke K., Bonab V.S., Yuan D., Manas-Zloczower I. (2018). Piezoresistive thermoplastic polyurethane nanocomposites with carbon nanostructures. Carbon.

[49] Feng C., Yi Z., Jin X., Seraji S.M., Dong Y., Kong L., Salim N. (2020). Solvent crystallization-induced porous polyurethane/graphene composite foams for pressure sensing. Compos. Part B Eng..

[50] Li X.P., Li Y., Li X., Song D., Min P., Hu C., Zhang H.B., Koratkar N., Yu Z.Z. (2019). Highly sensitive, reliable and flexible piezoresistive pressure sensors featuring polyurethane sponge coated with MXene sheets. J. Colloid Interface Sci..

[51] Zhong W., Ding X., Li W., Shen C., Yadav A., Chen Y., Bao M., Jiang H., Wang D. (2019). Facile Fabrication of Conductive Graphene/Polyurethane Foam Composite and Its Application on Flexible Piezo-Resistive Sensors. Polymers.

[52] Huang W., Dai K., Zhai Y., Liu H., Zhan P., Gao J., Zheng G.-Q., Liu C., Shen C. (2017). Flexible and Lightweight Pressure Sensor Based on Carbon Nanotube/Thermoplastic Polyurethane-Aligned Conductive Foam with Superior Compressibility and Stability. ACS Appl. Mater. Interfaces.

[53] Tewari A., Gandla S., Bohm S., McNeill C.R., Gupta D. (2018). Highly Exfoliated MWNT-rGO Ink-Wrapped Polyurethane Foam for Piezoresistive Pressure Sensor Applications. ACS Appl. Mater. Interfaces.

[54] Lv B., Chen X., Liu C. (2020). A Highly Sensitive Piezoresistive Pressure Sensor Based on Graphene Oxide/Polypyrrole@Polyurethane Sponge. Sensors.

[55] Zhao L., Qiang F., Dai S.-W., Shen S.-C., Huang Y.-Z., Huang N.-J., Zhang G.-D., Guan L.-Z., Gao J.-F., Song Y.-H. (2019). Construction of sandwich-like porous structure of graphene-coated foam composites for ultrasensitive and flexible pressure sensors. Nanoscale.

[56] Dong X., Wei Y., Chen S., Lin Y., Liu L., Li J. (2018). A linear and large-range pressure sensor based on a graphene/silver nanowires nanobiocomposites network and a hierarchical structural sponge. Compos. Sci. Technol..

[57] Ma Z., Wei A., Ma J., Shao L., Jiang H., Dong D., Ji Z., Wang Q., Kang S. (2018). Lightweight, compressible and electrically conductive polyurethane sponges coated with synergistic multiwalled carbon nanotubes and graphene for piezoresistive sensors. Nanoscale.

[58] Wu C., Huang X., Wu X., Qian R., Jiang P. (2013). Mechanically Flexible and Multifunctional Polymer-Based Graphene Foams for Elastic Conductors and Oil-Water Separators. Adv. Mater..

[59] Shu Y., Tian H., Yang Y., Li C., Cui Y., Mi W., Li Y., Wang Z., Deng N., Peng B. (2015). Surface-modified piezoresistive nanocomposite flexible pressure sensors with high sensitivity and wide linearity. Nanoscale.

[60] Wang Y., Zhu L., Mei D., Zhu W. (2019). A highly flexible tactile sensor with an interlocked truncated sawtooth structure based on stretchable graphene/silver/silicone rubber composites. J. Mater. Chem. C.

[61] Huang Y., Wang W., Sun Z., Wang Y., Liu P., Liu C. (2015). A multilayered flexible piezoresistive sensor for wide-ranged pressure measurement based on CNTs/CB/SR composite. J. Mater. Res..

[62] Karimov K.S., Ahmad Z., Khan M.I., Siddiqui K.J., Qasuria T., Abbas S.Z., Usman M., Rehman A.-U. (2019). Elastic layered rubber-graphene composite fabricated by rubbing-in technology for the multi-functional sensors. Heliyon.

[63] Giffney T., Bejanin E., Kurian A.S., Travas-Sejdic J., Aw K. (2017). Highly stretchable printed strain sensors using multi-walled carbon nanotube/silicone rubber composites. Sens. Actuators A Phys..

[64] Lee J., Kim J., Shin Y., Jung I. (2019). Ultra-robust wide-range pressure sensor with fast response based on polyurethane foam doubly coated with conformal silicone rubber and CNT/TPU nanocomposites islands. Compos. Part B Eng..

[65] Slobodian P., Riha P., Olejnik R., Matyas J., Kovar M. (2018). Poisson effect enhances compression force sensing with oxidized carbon nanotube network/polyurethane sensor. Sens. Actuators A Phys..

