# Supplementary material for: Preparation of Bamboo-Like Carbon Nanotube Loaded Piezoresistive Polyurethane-Silicone Rubber Composite
Source: Polymers (Basel). 2021 Jun 29;13(13):2144. doi: 10.3390/polym13132144 (PMC8272147; doi:10.3390/polym13132144)
Supplement: Supplementary file 1 [file polymers-13-02144-s001.zip › polymers-1243988-supplementary.pdf]

Preparation of Bamboo-Like Carbon Nanotube Loaded Piezoresistive Polyurethane-Silicone Rubber Composite

Mohammed Nabeel[1,2,a], Miklós Varga[1,b], László Kuzsela[3,c], Ádám Filep[4,c], Béla Fiser[1,5,e], Béla Viskolcz[1,f], Mariann Kollar[6,g], László Vanyorek[1,h,]

[1]Institute of Chemistry, University of Miskolc, 3515 Miskolc-Egyetemváros, Hungary

[2]Ministry of Science and Technology - Materials Research Directorate, Iraq

[3]Institute of Materials Science and Technology, University of Miskolc, 3515 Miskolc-Egyetemváros, Hungary

[4]Institute of Metallurgical and Foundry Engineering, University of Miskolc, 3515 Miskolc-Egyetemváros, Hungary

[5]Ferenc Rákóczi II. Transcarpathian Hungarian College of Higher Education, 90200

Beregszász, Transcarpathia, Ukraine

[6]Institute of Ceramics and Polymer Engineering, University of Miskolc, 3515 Miskolc-Egyetemváros, Hungary

[a]mohamedxl2006@gmail.com, [b]miklos568@gmail.com, [c]femkuzsy@uni-miskolc.hu, [d]femfilep@uni-miskolc.hu, [e]kemfiser@uni-miskolc.hu, [f]viskolcz@gmail.com, [g]femmaja@uni-miskolc.hu, [h]kemvanyi@uni-miskolc.hu

SUPPLEMENTARY INFORMATION

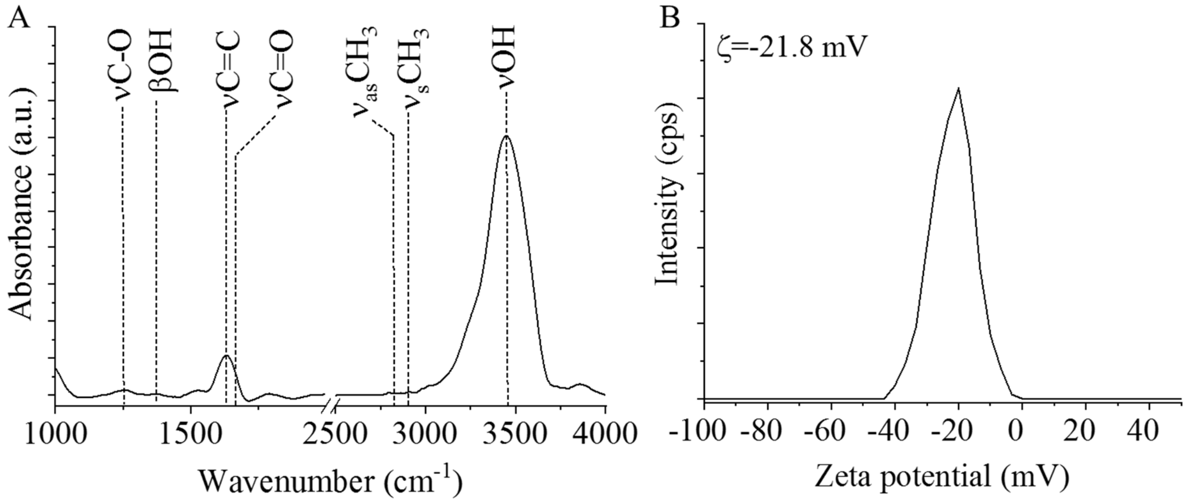

SI Figure S1. FTIR spectrum (A) and zeta potential distribution in aqueous dispersion (B) of the N-BCNTs.

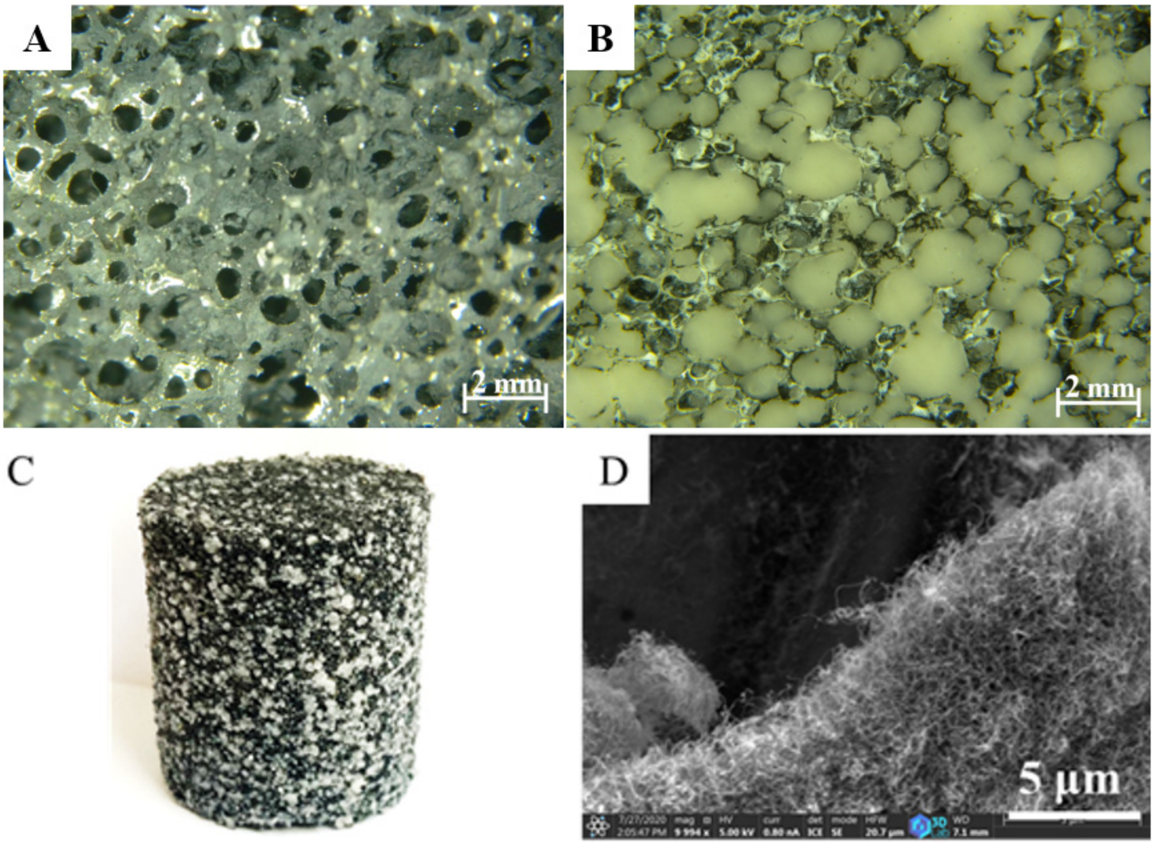

SI Figure S2. Optical microscopy images with 100x magnification of the surface of the pores in the PU foam covered with nanotubes (A) and the pores of the PU foam coated by N-BCNT and impregnated with silicone rubber (B). Photo of the silicon rubber loaded foam (C). SEM image of the N-BCNT coated polyurethane system (D).

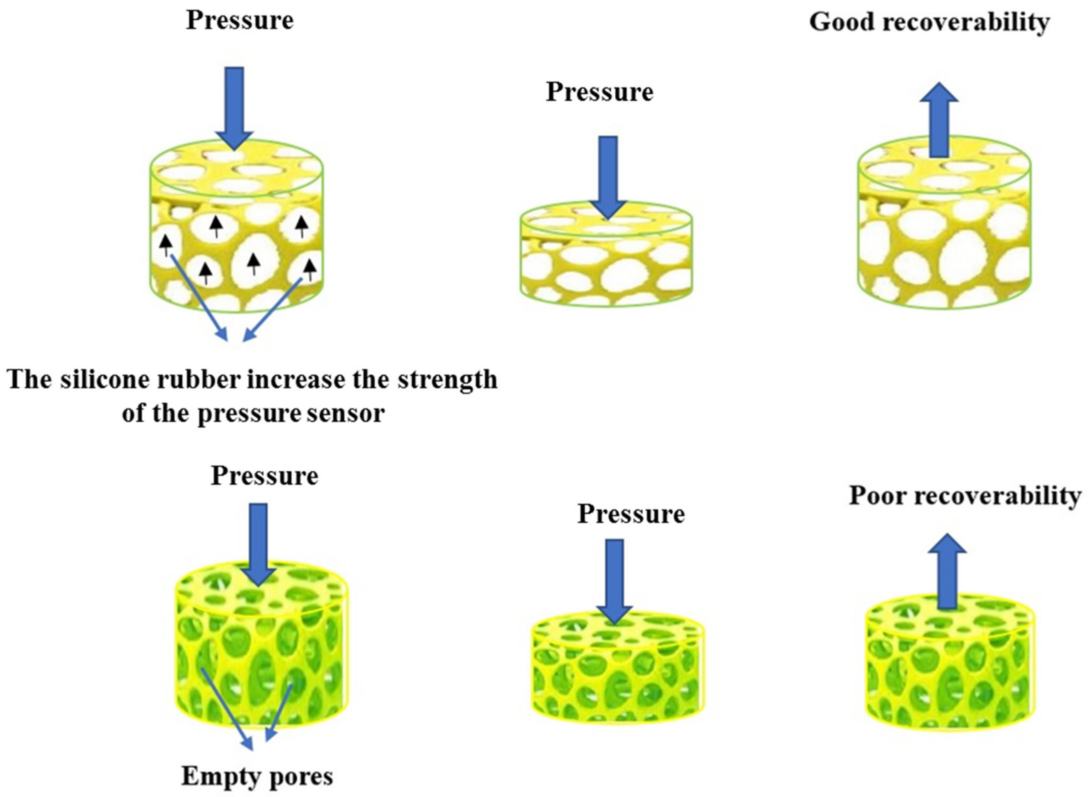

SI Figure S3. Schematic explanation of the effect of silicone rubber on the recoverability and strengthening of the prepared flexible piezoresistive nanocomposite, N-BCNT/PU@silicone rubber nanocomposite.

Further plans to produce N-BCNT/PU-silicone rubber nanocomposite in industrial scale

The industrial production of the designed system is under development. It is envisaged that a Topline casting machine (Hennecke GmbH) which is available at the Polyurethane Laboratory (University of Miskolc) will be used to produce the polyurethane foam. The equipment operates by a high-pressure process, and it is mainly suitable to produce soft polyurethane (PU) foam, but other types of PU systems can also be created. The catalyst and other additives are introduced into the system by pre-feeding into the polyol component of the raw materials. The mixing of the components (polyol and isocyanate) takes place in the mixing head by a counter current injection process. The robotic arm with a high-pressure mixing head (SI Fig. 4 A) injects the reaction mixture of the raw materials into the jacketed thermostat aluminium mould (SI Fig. 4 B). At the end of the process, the mould carrier opens, and the finished sample can be removed. The technology can be further extended to include additional steps (*e.g.* impregnation with carbon nanotubes, CNT) into the procedure. The PU foam produced by this semi-industrial process described above can be impregnated with an alcoholic dispersion of carbon nanotubes (as it was done in the laboratory experiments). After this step the PU has to be dried, and the foam could be returned to a vacuum injection mould within which the CNT containing foam can be loaded with liquid silicone rubber. This is a viable industrial scale procedure, but the design and implementation of the extended system which can be used to prepare the nanocomposite will be a lengthy mechanical engineering task.

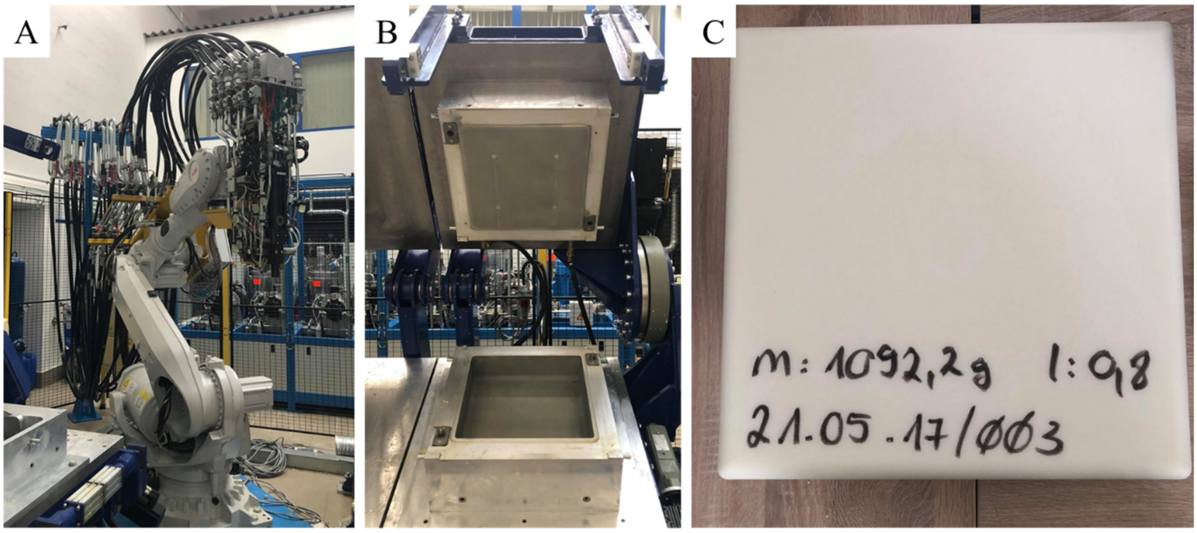

SI Figure S4. Robotic arm for the injection of PU components (A), the thermostat mould (B), and the as-prepared PU foam which is ready to be impregnated with N-BCNTs suspension (C)
